# Toward a safer school environment: A mixed-methods analysis of bullying and its impact on romanian middle school students

**DOI:** 10.1371/journal.pone.0340086

**Published:** 2026-01-05

**Authors:** Claudiu Coman, Anna Bucs, Angelica Banca, Marcel Iordache, Gabriela Motoi

**Affiliations:** 1 Transylvanian University of Brașov, Doctoral School of Social and Human Sciences of Craiova and the Academy of Romanian Scientists, Brasov, Romania,; 2 University of Craiova, Craiova, Romania,; 3 Vestern University of Timișoara, Timisoara, Romania,; 4 University of Craiova, Craiova, Romania; University of Luzon, PHILIPPINES

## Abstract

Our study investigated the complex and nuanced phenomenon of bullying in the Romanian educational context, using a mixed methods approach. Its objective was to analyze the implications and effects of bullying on students, the school environment, and school safety and to propose recommendations to elevate and enhance existing bullying prevention programs. The qualitative data was collected using the adopted Owelus Questionnaire and analyzed to determine the prevalence of bullying and its effects. Qualitative data was collected by conducting semi-structured interviews with different stakeholders such as teachers, parents, and experts who expressed their insights on the intricacies of this harmful and prevalent phenomenon in the Romanian educational context, their experiences, and possible risk factors. The results highlight a significant and negative effect on the emotional well-being of students. In addition, our research identified the different perceptions of the interviewed stakeholders: teachers, parents, and experts, highlighting the need for targeted and multiple interventions that include all key participants in the bullying phenomenon. This study suggests a better collaboration among teachers, parents, and the school community. Conclusions include recommendations for creating intervention programs that are comprehensive, specific, and efficient given the resources available to the Romanian educational system.

## Introduction

Bullying is a social phenomenon that erodes the fragile tissue of our society. Bullying in schools is a multifaceted phenomenon that reinforces a mix of psychological and sociological aspects. On one hand, the psychological implications aim to understand and solve the empirical manifestation of power imbalances between individuals or groups. On the other hand, the implications of sociology in deepening the subject stem from questioning the relationships between communities, the antagonisms created, and implicitly, the management of patterns in collective mentality.

Bullying must be seen as a complex and pervasive social phenomenon in all societies, regardless of their level of development, affecting a wide range of socio-economic categories of population and age groups.Of course, bullying and its forms of manifestation vary depending on the social and cultural context of a community, but on a global scale, it is recognized as a social problem because it is encountered in all societies (see, for example, the results of research conducted by UNESCO, 2019 or Save the Children) [1,2].

Another investigative path regarding the phenomenon arises from the emergence of clinical studies, from the medical assessment of behavioral transformations [3]. Because bullying is present both in real life and online, researchers and specialists in the social sciences draw attention to the estimated emotional impact, on behavioral degeneration in the case of both aggressors and victims.

Bullying represents a form of behavioral manifestation, individual or collective, that targets the vulnerable points of a person or a group of people. An aggressive relationship is established between the promoters of such behavior and victims through the language used or reactions. Most often, the targets of this social phenomenon experience various forms of violence, and the predisposition to general stigmatization inhibits potential reactions

Apart from existing literature regarding impact, prevention, and causes, bullying is a versatile concept associated in specialized literature with a wide range of definitions. The lack of consensus among specialists regarding standardizing a meaning stems from both the emergence of the phenomenon and its adaptation to new and evolving societal contexts.

Since our study focuses on the mixed analysis of bullying in schools in Romania, we consider it necessary to have a clear theoretical delimitation regarding the definitional trajectory. Thus, the work follows the logic proposed by Dan Olweus in defining the term focused on three primordial relationships: purpose, the existence of a power imbalance, and the generated harm [4]. From a sociological point of view, one definition of bullying that can be offered is that bullying can be defined as “repetitive, intentional aggression by an individual or group towards others perceived as unable to defend themselves” [5].

The manifestation of bullying in overlapping environments currently exacerbates its implications on victims. By overlapping environments, we refer to the alternating interactions in the physical and online environment, existing at the level of school communication. Technological advancements in contemporary society are associated with a series of interdependencies, but also with direct communication through devices. Thus, communication within school groups at the level of parents and students has the potential to exacerbate negative reports such as discrimination, stigmatization, or bullying.

In this study, we aim to analyze the complex phenomenon of bullying in Romanian middle schools from Craiova municipality. We applied the adopted Owelus questionnaire to collect quantitative data from students, evaluating the prevalence, types, and student perception. During the 1970s, Olweus coordinated the first large-scale studies on bullying and developed one of the first school bullying prevention programs, known as the “Olweus Program”, a program that is considered highly effective and is still used in schools around the world to reduce bullying in schools.

This program is based on the principle that bullying can be prevented and reduced through changes at the school level (here the role of decision-makers is extremely important) and by promoting harmonious relationships between pupils and is based on the following principles: awareness and involvement of the whole school community (pupils, teachers, decision-makers, parents), school-wide interventions, continuous training of teachers and teaching assistants in the identification and management of bullying behaviour [5].

In addition, we found from previous research that bullying is a nuanced, specific, and versatile phenomenon. In this regard, we conducted semi-structured interviews with parents, teachers, and experts to better understand and capture the complex characteristics of bullying in the Romanian educational system. By adopting such a mixed method approach, we propose to bring a significant contribution to understanding bullying among middle school students and also to offer recommendations based on results for developing an efficient strategy in bullying prevention and intervention programs.. In the Romanian educational context, where bullying remains unevenly reported and is shaped by cultural norms, institutional practices, and resource constraints, qualitative inquiry is particularly suited to capturing lived experiences and stakeholder perspectives that may not be fully reflected in quantitative indicators alone. The integration of qualitative data allows for a contextualized understanding of meanings, interpretations, and relational dynamics surrounding bullying, which is essential for informing culturally responsive prevention and intervention strategies. Such a comprehensive approach is needed to create a safe and positive learning environment to maintain the harmonious development of students

### Literature review

Bullying is an undisputed reality within school environments. The intensity of its manifestation or even the existence of distinct cases requires an understanding of the generating/aggravating factors, the behavior of the victims, as well as the aggressors. Furthermore, the level of severity of the associated consequences generates constant media coverage, increasing public attention to the phenomenon [6]. In the following section, we will analyze a correlation with specialized writings in order to highlight points of convergence at a theoretical level, as well as to evaluate the macro research orientations in existing literature.

The main descriptions of bullying as a phenomenon revolve around patterns: aggressive behaviors, distinct environments of occurrence, and the existence of power relationships marked by imbalances [7]. As early as 1996, the World Health Assembly highlighted violence as one of the main public health issues [8].

Some studies demonstrate that certain behaviors that fall under bullying tend to be prominent during lower education cycles [9]. Others argue that bullying peaks in middle school [10].

Many savants in the literature have conducted similar studies with the aim of understanding the bullying phenomenon. Lambe et al. [11] explore the intervention of colleagues in bullying incidents online and offline, using a socio-ecologic framework to better understand the impact of different influential factors for defense. This study adopted a mixed methods approach using questionnaires and interviews.

Evens and Smokowski [12] analyzed how ecological processes contribute to diminishing bullying in the academic sphere. Thomberg’s [13] study approaches bullying as a collective action, talking about stigmatization and fighting for identity. The methodology included interviews and observation to capture the complexities of social interactions that facilitate bullying. Gaffney et al. [14] conducted a meta-analysis to evaluate the efficacy of bullying intervention programs in schools on a global level, using data from a variety of studies to determine the impact of these programs.

Saldıraner & Gızır [15] conducted quantitative research: 20 interviews with principles from Mesin, Turkey. These defined a diverse structure of influencing factors such as family, personal, environmental, and teacher-related. The authors highlight the nuanced nature of this phenomenon. They argue that understanding the motivating factors of bullying, school leadership, social support, and the placement of effective prevention programs are critical in developing a safe learning environment for middle school students.

Mischel and Kitsants [16] adopted a mixed methods approach using questionnaires and interviews. They argue that those students who were victims of bullying declared being involved in more bullying incidents. They also show that a negative learning environment was liked strongly, with high bullying incident rates. In addition, the qualitative data offers valuable insights into the individual experiences of each interviewed student. They highlight, in essence, the importance of social support in combating bullying.

Juvonen and Graham [17] explore the complex dynamics of bullying in schools, focusing on both the power wielded by bullies and the severe consequences suffered by victims. This detailed study analyzes the characteristics and motivations of bullies, the effects on victims, and the role of the school environment in perpetuating or preventing bullying. Bullies, according to the research, are not a homogenous group, with Juvonen and Graham emphasizing that bullies can range ‘from popular students who use bullying to maintain their social status to those who have emotional problems and use bullying as a form of expressing frustration’ [17].

Juvonen [18] investigates the emotional implications of body weight stigmatization among middle school students, focusing on weight-based peer discrimination. The analysis included a sample of 5,128 young people from 26 urban schools, exploring how weight-based discrimination influences adolescents’ mental health, and the results highlighted ‘significant links between weight stigma and emotional problems such as depression and anxiety’ suggesting the need for school-based interventions to combat these stigmatizing attitudes.

Vaillancourt, Hymel & McDougall, [19] examines why and how bullying has lasting effects throughout a person’s lifetime. According to the study’s authors, victims of bullying are at higher risk of developing mental and physical health problems that can persist throughout their lives. These include depression, anxiety, sleep disorders, and problems with memory functioning.

Strindberg, Horton, & Thornberg [20] examine students’ perspectives on victimization and the role of the witness in bullying situations and explore students’ fears and experiences of being the target of bullying, 79 as well as the dynamics that lead them to act or not act when they witness bullying. Through the analysis of interviews and focus groups, the paper provides a detailed understanding of the factors that influence students’ behavior in such situations, highlighting the complexity of their experiences and the significant impact that bullying has on school life [20]. The study also shows that while many students recognize the injustice of bullying, fear of retaliation discourages them from taking action.

In another study, Huang&Cornell [21], show that “bullying rates can vary significantly by sociopolitical context”, exemplifying how “external factors can influence bullying behavior in schools” [21]. The main finding of the research was that in US localities where the majority of voters favored a particular political candidate, bullying rates were higher. The authors suggest that some behaviors of political leaders may serve as role models for the general population, including young people.

Álvarez-Guerrero, et al., [22] conducted a study that provides a detailed analysis of the effectiveness of dialogic meetings in preventing bullying among students with special education needs. What is interesting about this study is that it presents the results of an intervention method, based on the Dialogic Gatherings (DG) technique, in two elementary classes with a total of 43 pupils, 5 of whom had special educational needs (SEN). The interventions helped to increase students’ awareness of violent and non-violent behaviors, they began to prefer non-violent behaviors and to better understand the differences between abusive and healthy relationships, therefore the DG method “effectively contributed to increasing student’s awareness of the distinction between violent and non-violent relationships” [22]. Another research finding presented in the study was that building a safe and supportive learning environment through dialog-based interventions was essential for the inclusion of students with special educational needs, particularly because “inclusive learning environments are essential to ensure that all students, including those with SEN, feel included and valued” [22].

Hoareau, Bages & Guerrien [23] investigate how adolescents use information and communication technologies (ICTs), such as the Internet and mobile devices, and the relationship between their use, self-control, and engagement in cyberbullying or cyber victimization. The main findings of the research indicate a significant relationship between lack of self-control and the tendency to engage in cyberbullying. The study also showed that internet addiction is strongly associated with the risk of becoming a victim of cyberbullying. The study uses self-reported questionnaires completed by a sample of 264 French adolescents, ranging in age from 11 to 15 years, to assess the frequency of internet use, type of devices used, locations of internet use, range of activities engaged in online, as well as the degree of internet addiction and levels of self-control.

### Bullying in Romania

The phenomenon we are investigating is present in Romania through the Anglo-Saxon term “bullying” [24]. It refers not only to school environments in which bullying is projected but also to other contexts in which the bully-victim relationship is systematized.

In Romania, bullying is intensively studied, especially due to the identification of differentiated variables regarding the experiences and interactions among students. Some results mark a correlation between aggressive behaviors and the existence of conflicts in school environments [25]. Others center their expertise around the impact of mediation, the professionalism with which certain teachers react to flatten the phenomenon’s existence. Elements such as verbal or physical aggressiveness, the process of stigmatization, or even the lack of control over reactions, all build the premises for the prevalence of bullying in Romanian schools.

Gavrilescu and Merloiu [26] argue that bullying is a major issue in the Romanian educational context, and it affects not only aggressors and victims but also witnesses and the entire school environment. The authors also suggest intervention programs within the family and the school context, such as open communication, active participation in school life, and a zero-tolerance rule towards aggression.

Tomescu [27] suggests that the Romanian context poses multiple challenges because of the high prevalence rate and effects on student’s mental state. They found the biggest issue is verbal bullying because teachers do not respond adequately to these behaviors. There is a lack of resources and training regarding bullying prevention initiatives. The phenomenon is culturally specific and complex. This is why our objective is to explore the craiovean area of the country to further contribute to existing literature and to understand this consistent issue in our educational system.

Mureșan and Stan [28] researched bullying in Cluj by addressing students on their opinion regarding bullying prevalence and their perception of this phenomenon. They also found that verbal bullying was the most prevalent (45%). They also found that bullying is significantly linked to feelings of depression, anxiety, loneliness, and thoughts of suicide [28].

Similar to the authors above, Drăghicescu and Stăncescu [29] utilized research conducted by the Save the Children Organization in 2016. They highlight the fact that teachers are cautiously aware of the magnitude of this phenomenon’s negative effects on the school environment and student’s mental health. However, they do their best with the resources they have available, such as promoting a positive learning environment and communicating with parents, but a more detailed understanding of the teacher’s perspective is needed to fully comprehend the bullying phenomenon in Romania.

Niță [30] argues that to prevent bullying more efficiently, there needs to be some systematic changes, such as a clear definition of bullying and clear legal sanctions. They need to be executed depending on the gravity of their effects on victims. In addition, they argue that teachers should be trained in this manner to combat bullying more efficiently.

G.V. Bonea from the Quality of Life Research Institute, Romanian Academy, addressing aggression and violence in Romanian schools. The study published in the Quality of Life journal aims to identify and analyze the “phenomenology and causality of aggression and violence in schools” [31], in order to discover new and effective ways to prevent and combat this problem. It emphasizes the need for an effective and sustainable response, involving governmental, institutional, social, school, and family efforts. begins by defining aggression and violence in the school context, highlighting the need to differentiate these behaviors and address them through appropriate educational and social policies.

Another study, developed in 2018, aims to investigate teachers’ perceived severity of three types of bullying: physical, verbal, and social exclusion. The author of the study also analyzes teachers’ responses to bullies and victims involved in each type of bullying behavior [32]. Therefore, the study reveals that teachers perceive ‘verbal bullying as more severe than physical and social bullying’, the results are similar to other international studies. Young and less experienced teachers are more likely to perceive physical bullying as more severe. According to the literature, “the perceived severity of bullying behavior is one of the strongest predictors of teachers’ response to bullying” [32].

### Themes and research gaps

Based on our literature review we, the authors identify the following emergent themes across existing literature:

- The complexity of bullying: most studies highlight the nuanced and multifaceted nature of the phenomenon, including various forms of aggressive behaviors such as physical, verbal, and cyberbullying. In addition, savants argue that motivators of such behaviors are power imbalances, social status, and frustration. In consequence, bullying causes distress, negative emotions, problems with academic performance, and social isolation.
- Impact on mental health: A prevalent theme among researchers is the harmful and negative impact bullying has on well-being and mental health. Studies argue that bullying victims report high stress and anxiety levels, depression, and low self-esteem.
- Benefits of mixed-method approaches: Savants are more and more open to a mixed-methods approach to get a more comprehensive understanding of bullying.
- The school environment: In most of the studies, the school environment had a pivotal role, and researchers highlighted the importance of promoting a positive learning space by training teachers and managing bullying effectively. The importance of school policies, peer groups, and teacher responses are most frequently mentioned.

The themes above highlight the need for a specific and contextual approach to bullying because of its complexity. The negative effects on mental health further emphasize the need for effective intervention programs focused on this aspect. In addition, researchers suggest a mixed-method approach to comprehensively evaluate the phenomenon of bullying. Finally, the literature suggests a targeted approach to intervention programs that include all important stakeholders in bullying. Based on these needs, we formulated our research questions.

## Methodology

Our study aimed to analyze the complex phenomenon of bullying and its effects on middle-school students in the urban areas of Craiova municipality. We adopted a mixed-method approach to capture the intricacies of this specific phenomenon, which varies contextually and culturally.

We analyzed schools from the urban area specifically, and the data collection period started on the third of May 2023 and ended on the 28th of June 2023. It had two stages. In the quantitative data collection stage, we applied the adopted Save the Children Romania Questionnaire face to face. The qualitative sample consisted of 673 subjects of students from five to eight graders. The sample was selected by a stratified convenience method.

Our data collection period included applying the questionnaires face-to-face, and we, the authors, respected GDPR measures and all of the subjects’s identities. Both quantitative and qualitative data have been treated in this way. The Council of the Sociology and Communication Faculty has approved our study—number: 22 Date: 15.03.2023. We obtained written participant consent.

### The research instruments

The research instrument for the quantitative part was adapted from the sociological questionnaire used by Save the Children Romania for the research “Bullying among children. Sociological study at a national level” [33]. Also, some of the response options for question 31 were adapted from the “Olweus Bully/Victim Questionnaire.”

The questionnaire for the research on bullying among pupils included several sections: a section that focused on socio-demographic data (in order to be able to correlate these variables with the answers to some questions), questions about the family the pupils belonged to, questions that targeted bullying situations in which they found themselves (either as victim, bully or witness), questions about how safe is the school they attend, etc. We also asked indirect questions, which allowed us to find out how the pupils felt about bullying and especially its effects.

The questionnaire that we used to be applied to pupils from secondary schools in Craiova was adapted according to nationally and internationally validated instruments, such as the questionnaire used by Save the Children Romania and the “Olweus Bully/Victim Questionnaire”.

The questionnaire was adopted after the instruments were validated. It included questions about students’ socio-demographic backgrounds, bullying experiences, perceptions of the school environment, and relationships with parents and teachers. The questionnaire included five open-ended questions, 15 close-ended questions, and 10 items with a Likert Scale. The collected data was then processed and analyzed by IMB SPSS version 23, using descriptive statistics, Pearson Correlations, Qhi-Square, and ANOVA tests to understand the correlations between variables. The questionnaire was tested in a pilot study, and it showed consistent results with a Cronbach Coefficient of α = 0.85.

We also used the interview-based opinion survey method to test some of the hypotheses of our research using techniques other than the questionnaire and to complement the quantitative data and obtain detailed and nuanced information. This qualitative method involved conducting semi-structured interviews with three categories of respondents: parents, teachers, and experts (psychologists, school counselors, school inspectors, police representatives, social workers, etc.).

To realize the qualitative research, we applied the three interview guides to 77 people, who were distributed as follows: 38 parents of secondary school students, 17 experts from different key fields related to bullying – school counselors, social workers, psychologists, representatives from the Police, Gendarmerie, social directors, school inspectors, and 22 interviews were applied to teachers in pre-university education, who teach at secondary school level. The recruitment period started in parallel with the qualitative data collection period, from the third of May 2023, and ended on the 28th of June 2023. At this stage, all necessary data has been collected.

The interviews were guided by well-defined structures for each category of respondents, but there were also common questions, which were found in each of the interview guides we used:

Interviews with parents: the interview guide included questions about parents’ perceptions of bullying, the prevention and intervention measures they consider effective, and their role in educating their children about bullying.

Interviews with teachers: The questions focused on understanding the phenomenon of bullying in the school environment, school strategies and policies for prevention and management, and evaluating their effectiveness.

Expert interviews: The interview guide included questions about factors contributing to bullying, the effectiveness of prevention programs and the need for additional resources to support victims of bullying.

These subjects were selected using directed sampling to obtain diverse perspectives and experiences. The interviews were structured around the concept of bullying and its definition, personal experiences with bullying, its effects on students, and efficient prevention suggestions.

These interviews were then transcribed, and identifying patterns and themes began, allowing a detailed and nuanced understanding of the phenomenon in the Craiovean context. We used three interview guides for parents, teachers, and experts. Each one had between 15–20 semi-structured questions focusing on the following themes: perception of bullying, prevention strategies, and the role of social and parental support.

Although we used three different interview guides, there were also common questions in the interview guides for teachers, experts and parents, for example, the question What do you understand by bullying and what are the different forms it can take in the environment educational setting? Or What are the factors that contribute to bullying in schools? We opted to use these common questions in order to be able to observe the different or common ways in which teachers, experts and parents approach and understand the problem of bullying, which was very useful for us to be able to construct the set of recommendations for preventing and combating bullying that we will present in the conclusion part of this paper.

All participants from both stages of data collection were informed about the purpose and aim of our study. Subjects’ privacy and confidentiality were respected throughout the data collection period. Our mixed-method approach allows a comprehensive understanding of the bullying phenomenon in the Romanian educational context.

### Study limitations

Our study sample of 673 students was representative of Craiova. However, it did not take into account the urban areas, which makes the generalization of the results quite difficult. Moreover, subject responses were based on self-reporting evaluations, which can impact the collected data. To ensure the validity of data collected by reviewing questionnaires, double coding of data, meaning that two individuals conducted the interviews, allowing two perspectives to be compared. While a formal member-checking procedure was not conducted, interpretive validation was supported through feedback obtained during and after the interviews, as well as through triangulation across stakeholder groups and collaborative coding among the research team. Further research must be conducted in order to have a comprehensive understanding of bullying in the Romanian educational context.

In addition, asking the students about their past experiences could indicate recall bias, meaning that subjects may not remember correctly the frequency or the intensity of their experiences.

Further research should also focus on longitudinal effects because this present paper provides data from a specific and short period. A longitudinal study could offer valuable information about the effects of bullying over time.

### Study results

This study’s main objective was to analyze the complex phenomenon of bullying in the Romanian educational context. To that end, we, the authors, created the following hypotheses and research questions.

Hypothesis 1: The more frequently students are exposed to bullying, the more likely they are to report symptoms of stress and anxiety.

Hypothesis 2: If students perceive bullying as something that occurs frequently and has negative effects, then they will report lower school satisfaction.

Research question 1: The ways of reporting bullying (frequency, severity, types of aggressive behaviors) differ significantly between students, parents and teachers, reflecting different perceptions and interpretations of the phenomenon.

Research question 2: Experiences of bullying affect pupils’ social relationships, leading to increased social isolation.

Research question 3: The more parents and teachers perceive that anti-bullying measures in schools are insufficient, the more one of the proposed measures will include more rigorous prevention and countermeasures based on monitoring and tougher sanctions.

In the following section of this paper, we present the results relevant to our main objective and research questions.

The problem of bullying remains difficult to identify in Craiova schools because many students choose not to report it. To our question, 25.3% of the students indicated that they had not shared their experience with anyone, we believe that this is out of fear of not being understood, fear of being judged, and out of shame. There is also a percentage of 39.8% of the students that caught our attention because they indicated that they were “not sure”, again, this could be students for whom there is a possibility that they have been bullied and hesitate to give a clear answer to this question also for fear of being judged or because of shame.

In our research, we were interested in observing whether there is a difference between genders in terms of sharing cases of bullying and for this, we conducted a statistical analysis by Chi-Square test between the variable gender and the answers to the question that was aimed at sharing your bullying experience. The processing that we carried out through the SPSS program, which is presented in Table 1, revealed that there is a significant gender difference in sharing the experience of bullying.

**Table 1:** Result of Chi-Square Test between the two variables.

	Value	df	Asymptotic Significance (2-sided)
Pearson Chi-Square	12,281^a	2	,002
Likelihood Ratio	12,323	2	,002
Linear-by-Linear Association	10,120	1	,001
N of Valid Cases	619		

a.0 cells (0,0%) have expected count less than 5. The minimum expected count is 76,36.

**Source: Own processing, SPSS.**

**Are students who have been exposed to bullying more likely to report symptoms of stress and anxiety?**

**Theme: negative emotional effects of bullying (anxiety, stress, isolation, behavioral changes, depression)**

**Parent’s perspective:**

When we conducted the qualitative data collection there was a lot of talk about the aggressive side of bullying and the intentional manifestation of this behavior. At the same time, some parents also indicated the negative consequences that this type of behavior can have on their children, and most of them mentioned the severe emotional impact, such as low self-esteem, anxiety, and depression. ”Bullying increases the risk of depression, anxiety, sleep difficulties, poor academic performance, and even school dropout (B.C., F, 53, economist)”.

One of the interviewed parents mentioned that she noticed at a certain point that her son became sad and irritable in his relationships with other children, after being the target of bullying, and this behavior change was a clear signal that something was wrong. ”Yes, my son has been, on several occasions, the target of bullying acts by other classmates in school, due to a visible physical disability.. I noticed a change in his behavior, becoming sad and irritable (M.C.P., F, 45, university assistant)”.

**Expert’s perspective:**

The consensus among experts is that bullying has a profound impact on the emotional and psychological development of its victims and that recognizing and clearly defining this behavior is essential for the implementation of appropriate prevention and intervention measures.

”Bullying has serious consequences on the victim, both emotionally and academically (E.M.)”.

”Bullying is a repeated and intentional behavior through which the aggressor harms, persecutes, and intimidates the victim (M.P., 38 years old, school counselor)”.

”Lack of supervision and involvement in activities by teachers and parents contribute to the occurrence of bullying in schools (C.A.)”.

**Teacher’s perspective:**

Some teachers confessed that the students they worked with at the class level became shy, withdrawn or, on the contrary, agitated, although they had not previously exhibited such behavior, and in their opinion, these are clear symptoms of involvement in a bullying incident. There were also teachers who mentioned that they noticed a decrease in self-esteem in some of their pupils. ”The student is sadder and more apathetic than usual. There is a decrease in self-image and self-esteem (D.S., 47 years old, primary school teacher)”.

After performing the statistical calculation for Pearson (Table 2) correlation, a value of −0.186 was found between involvement in bullying incidents and perception of school safety: when students’ involvement in bullying incidents increases, the perception of general safety in the school they attend decreases. Furthermore, we can observe from the above table that a Sig. (2-tailed) of 0.000, which means that this correlation is strong and statistically significant.

**Table 2:** Correlation between bullying incidents and perception of school safety and climate.

		21. Did you ever experience involvement in a bullying incident at school?	17. Is the overall climate of safety in school perceived as positive?
21. Did you ever experience involvement in a bullying incident at school?	Pearson Correlation Sig. (2-tailed) N	1 664	−.186** .000 656
17. Is the overall climate of safety in school perceived as positive?	Pearson Correlation Sig. (2-tailed) N	−.186** .000 656	1 662

** Correlation is significant atb the 0.01 level (2-tailed).

**Source: Own processing, SPSS.**

Students who responded to our questionnaire indicated that the bullying incident in which they were involved did not significantly affect their relationship with school and learning (Table 3). In the case of 9.6% of the pupils, the bullying incident made them not want to go to school so much, for this reason, we consider that some of the strategies to identify cases of bullying should be based on the analysis of absenteeism and dropout. This percentage of 9,6% correlates also with the 10,7% of the respondents who indicated that after the bullying incident, they started to see school as an unsafe environment, from our point of view.

**Table 3:** How do you think the bullying incident has affected your relationship with school and learning?.

		Frequency	Percent	Valid percent	Cumulative percent
Valid	Other	89	13,2	13,9	13,9
	No significant impact	381	56,6	59,7	73,7
	Affected my concentration at school	39	5,8	6,1	79,8
	Made me feel like I didn’t belong at school	61	9,1	9,6	89,3
	Made me feel unsafe in the school environment	68	10,1	10,7	100,0
	Total	638	94,8	100,0	
Missing System		35	5,2		
Total		673	100,0		

**Source: Own processing, SPSS.**

Ultimately, they are the ones who end up truanting and dropping out of school. 6.1% of our respondents indicated that following the bullying incident they had difficulty concentrating at school and had poor school performance (this percentage contributes to validating our second research hypothesis).

From the above table (Table 4), it can be seen that the estimated mean correlation is −0.185, indicating a moderate negative correlation between the perception of school safety climate and involvement in bullying incidents, which means that students who perceive the school as less safe are more likely to be involved in bullying incidents.

**Table 4:** Correlation between school climate and bullying incidents.

			17. How do you appreciate the climate of general security in the school?	21. Have you ever been involved in an incident of bullying at your school?
17. How do you appreciate the climate of general security in the school?	Posterior 95% Credible Interval N	Mode Mean Variance Lower Bound Upper Bound	662	-,186 -,185 ,001 -,258 -,111 656
21. Have you ever been involved in an incident of bullying at your school?	Posterior 95% Credible Interval	Mode Mean Variance Lower Bound Upper Bound	-,186 -,185 ,001 -,258 -,111	
	N		656	664

a.The analyses assume reference priors (c=0).

**Source: Own processing, SPSS.**

**The ways of reporting bullying (frequency, severity, types of aggressive behaviors) differ significantly between students, parents and teachers, reflecting different perceptions and interpretations of the phenomenon.**

**Theme: a different perspective, the tendency to underestimate bullying, complex phenomenon**

**Student’s perception:**

Regarding qualitative data, we found a difference in how students, parents, and teachers perceive bullying. After conducting our interviews, we observed that students find the bullying phenomenon severe with considerably negative effects on their mental state and overall well-being. Many of them indicated having symptoms of stress, anxiety, and low levels of self-esteem. Students also argued that they have lower school satisfaction when they are being bullied.

**Parent’s perception:**

In contrast, parents tend to underestimate the complexity and gravity of the effects bullying has not only on victims but also on the overall school environment and learning space. They consider bullying to be part of the socialization process, and they often think that students exaggerate when talking about their experiences. This perception can lead to them neglecting the severity of their children’s situation.

**Teachers’s perception:**

Teachers have a more accurate and deeper understanding of the dynamics and effects of bullying. Some do not agree with the frequency that students report. They recognize the negative effects on academic performance but tend to forget about the long-term effects of this phenomenon.

These differences in perceptions of the stakeholders highlight the necessity of a program that brings awareness to the realities of bullying, one that involves all essential participants of this phenomenon. This confirms our research question.

**Expert’s opinion:**

Each of the experts to whom we applied the interview guide brought up the need for legislation to combat bullying and support the effects of bullying more effectively. One of the teachers mentioned that the organization of information and awareness campaigns in schools should be a mandatory activity, to be organized at least annually, if not every semester.

On the other hand, he pointed out that the law that would sanction bullying should be stricter and based on clearer sanctions for bullies. Another teacher talked about how important it is to have collaboration between different key actors in this issue and this collaboration should also be mentioned in a legislative document.

There have been teachers who have signaled that many activities could be done in this regard if there were funds allocated for this, and one of the measures that would be taken, in the hypothetical situation of the Minister of Education would be to allocate sufficient funds for the prevention and combating bullying at all levels of education. ”Legislation should include clear sanctions for bullying behaviors to deter such actions (S.C., 38 years old, teacher)”.

”Mandatory education and awareness programs should be implemented in all schools (C.S.D., 49 years old, history teacher)”.

”Victims and bullies should receive support and counseling to manage trauma and prevent recurrence (N.G.A., 40 years old, teacher)”.

”Close collaboration between schools, police, social services, and other institutions is important to effectively manage bullying cases (VIL.L., 48 years old, geography teacher)”.

As seen in the previous pages, counseling was mentioned quite often by teachers as part of a strategy to combat bullying. In our interview guide, there was also a question on the role of the school counselor, because we wanted to find out more about how teachers perceive this role. In the view of our respondents, counselors are the most able to provide students, especially those who are victims of bullying, with the emotional support they need. The counseling techniques they apply at the counseling office, which can be individual or group counseling, were mentioned as very useful because they help the victimized students to better manage the stress and anxiety they are experiencing as a result of the incident.

”Students who are victims of bullying can be offered individual or group counseling to express their feelings and learn stress and anxiety management strategies (V.L.L., 48 years old, geography teacher)”.

”Organizing support groups and mentoring for bullying victims can provide a safe and supportive environment (N.G.A., 40 years old, teacher)”.

”Participation in personal development activities can help victims develop emotional management skills and positive relationships (D.L., 35 years old, teacher)”.

**Do bullying experiences affect pupils’ social relationships, leading to increased social isolation?**

**Theme: negative emotional effects of bullying, shame, humiliation, pain, isolation, suffering.**

**Expert’s perspective:**

Suffering is mentioned by several experts as a direct consequence of bullying, reflecting the deep and painful emotional impact on the victims: suffering, bully, victim. Also, humiliation is identified as one of the main feelings experienced by victims of bullying, indicating the effect of degradation and loss of self-respect: humiliation, pain, and shame. Another respondent describes the state of continuous anxiety and fear in which victims of bullying live through three words: sadness, fear, and depression.

”Bullying is any form of verbal or non-verbal aggression perpetrated by one child or multiple against another child/children (M.C.P., F, 45, university assistant)”.

”Bullying can lead to the victim’s isolation, lack of self-confidence, and poor academic performance (A.M., F, 48, dentist/university professor)”.

”Bullying is an aggressive and intentional behavior that is repeated between children or adolescents (M.S., M, 47, tourism operator)”.

”Bullying involves a power imbalance between two individuals, most often children, and can have dangerous consequences on normal development (B.C., F, 53, economist)”.

**Parent’s perspective:**

Parents have also warned us that social withdrawal and isolation are clear signs of bullying. For example, children who are victims of bullying become withdrawn from social situations and give up activities they previously enjoyed. ”A child victim of bullying will isolate themselves from others, lose self-confidence, have poor academic results, and may become an aggressor themselves. This tendency towards isolation can have long-term consequences on the child’s social development (A.M., F, 48, dentist/university professor)”.

The words indicated by the majority of the respondents also confirm the psycho-sociological theories that we have presented in the theory chapters of this doctoral thesis, which emphasize that bullying has extremely strong effects on the victims, especially of a psychological nature, effects that can remain in the long term, and in the case of secondary school students, can influence their future adult life. Based on the 12 interviews, we analyzed the extent to which the identified codes appear in each interview so that we could create a graphic matrix that clearly shows what bullying means in the vision of our respondents. The matrix highlights that in the definition of bullying, the keywords (most frequently used by the respondents) were: aggression, humiliation, pain, and fear. Therefore, aggression is a frequently mentioned code, being present in more than one respondent; bullying and victimization are other frequently mentioned codes, underlining the coercive nature and impact on victims; suffering and pain also appear in the responses of several experts, indicating the deep emotional impact of bullying. Codes such as fear, frustration, and poverty appear less frequently but are significant for understanding the diversity of perceptions of bullying.

Social bullying involves excluding or isolating a student from groups, spreading rumors, and denigration. These forms of bullying have a significant impact on victims’ self-esteem and sense of belonging. ”In my opinion, the most common forms of bullying are verbal and social bullying.

Social bullying almost always accompanies other types of bullying, with the victim student being excluded from the group or from various activities (E.M., 48 years old, NGO project manager)”.

**The more parents and teachers perceive that anti-bullying measures in schools are insufficient, the more one of the proposed measures will include more rigorous prevention and countermeasures based on monitoring and tougher sanctions.**

**Theme: insufficient measures, strict monitorization, collaborative efforts.**

This research question was confirmed by the qualitative interviews with parents and teachers, which revealed a common perception that current prevention and intervention measures are insufficient. Many respondents called for more rigorous and effective strategies, including awareness-raising programs, teacher training sessions, and direct interventions for affected pupils. These demands reflect a growing awareness of the need for more proactive educational policies to tackle bullying.

**Parent’s perspective:**

The measures for good management of bullying that parents consider effective are related to the family and the school where the child is educated, where educational programs should be put in place to raise awareness of the problem among students and teachers. One of the parents told us that these programs can help students acquire self-control, conflict negotiation, communication, etc., but the same skills would also be useful for teachers. ”I would organize programs to inform students about the proper ways to handle specific bullying situations, focusing on developing competencies in understanding and self-control, conflict negotiation, communication, and self-defense means (G.C.L., F, 45, architect)”.

In the view of another parent, the school should implement a more rigid system of monitoring children’s activity, referring to the installation of surveillance cameras in areas where there is a risk of bullying incidents, and ensuring the presence of an adult (teacher on duty/local police officer), permanently, during recess, especially in the school toilets (D.M.D., F, 43 years old, clerk). Beyond these measures, most of the parents again referred to a framework of regulations to be strictly enforced, containing well-defined intervention procedures and types of sanctions appropriate to each aggressive behavior. For some parents, bullying cases persist in schools because they are not reported urgently. After all, pupils do not communicate with teachers and sometimes not even with parents. „There is a need to create intervention procedures in school bullying and to continuously monitor high-risk bullying areas using video surveillance systems (G.C.L., F, 45, architect)”.

”Bullying attempts should be reported promptly and given extraordinary attention, analyzing them carefully, summoning the involved students and their parents to the school (S.A.C., F, 36, court clerk)”.

**Experts’ perspective:**

They confirm that victims of bullying are often reluctant to talk about their experiences out of fear or shame, which makes timely identification and intervention difficult. Without a clear and coherent framework, interventions are often piecemeal and ineffective, and because disguised forms of bullying, which are more subtle and difficult to identify, are a significant obstacle:

”Victims’ reluctance to talk about their experiences out of fear or shame (N.L.L., 48 years old, deputy director)”.

”The lack of a clear and consistent anti-bullying policy, the lack of effective mechanisms for reporting bullying incidents (B.T., 48 years old, school inspector)”.

”There are masked forms of bullying that are difficult to identify. There is no functional system for identifying and punishing the aggressor (E.M., 48 years old, NGO project manager)”.

Ongoing training for teachers and school staff is important to ensure that all those involved are up to date with the latest methods and strategies for bullying prevention and intervention. Educational resources such as information materials, workshops, and information sessions were also mentioned as necessary to educate pupils, parents, and school staff about bullying and ways to prevent and intervene.

”Continuous training in the field of bullying is important to better understand the phenomenon and acquire effective skills and strategies to combat it (C.A., 47 years old, school principal)”.

”Workshops for parents on bullying behaviors and intervention methods, involving them in school administration councils to develop school policies and programs. Parents can be partners in monitoring their children’s behavior and providing support and guidance in difficult situations (J.F., 41 years old, officer, Ministry of National Defense)”.

**Teacher’s perspective**

Each of the teachers to whom we applied the interview guide brought up the need for legislation to combat bullying and support the effects of bullying more effectively. One of the teachers mentioned that organizing information and awareness campaigns in schools should be a mandatory activity, to be organized at least annually, if not every semester. On the other hand, he pointed out that the law that would sanction bullying should be stricter and based on clearer sanctions for bullies. Another teacher talked about how important it is to have collaboration between different key actors in this issue, and that this collaboration should also be mentioned in a legislative document.

”Legislation should include clear sanctions for bullying behaviors to deter such actions (S.C., 38 years old, teacher)”.

”Mandatory education and awareness programs should be implemented in all schools (C.S.D., 49 years old, history teacher)”.

”Victims and bullies should receive support and counseling to manage trauma and prevent recurrence (N.G.A., 40 years old, teacher)”.

”It is important to have close collaboration between schools, police, social services, and other institutions to effectively manage bullying cases (VL.L., 48 years old, geography teacher)”.

There have been teachers who have signaled that many activities could be done in this regard if there were funds allocated for this, and one of the measures that would be taken, in the hypothetical situation of the Minister of Education would be to allocate sufficient funds for the prevention and combating bullying at all levels of education.

## Discussion

The main aim of this paper was to investigate the phenomenon of bullying in secondary schools in Craiova, addressing its perceptions and its impact on students. This localized approach allows the identification of regional and cultural particularities of the phenomenon, which may influence the dynamics of bullying and the effectiveness of intervention measures. The research revealed that bullying is a widespread and diversified phenomenon in the schools analyzed, manifesting itself in multiple forms. This diversity underlines the phenomenon’s complexity and the need to approach it from a multidimensional perspective. The quantitative and qualitative research confirmed that bullying has significant negative effects on the emotional state and academic performance of victims. Affected students report increased levels of anxiety, depression, and lowered self-esteem, which negatively influence their school and social life. An innovative aspect of the thesis is the focus on students, parents, and teachers’ differentiated perceptions of bullying. This allows for a more nuanced understanding of how each group perceives the seriousness and frequency of bullying, highlighting discrepancies and potential barriers in communication and intervention.

Hypothesis 1: The more frequently students are exposed to bullying, the more likely they are to report symptoms of stress and anxiety. This hypothesis was confirmed by the data collected, which showed a significant correlation between the frequency of exposure to bullying and the increased level of stress and anxiety symptoms reported by students. Statistical analysis, including Pearson and ANOVA tests, indicated that students who had experienced repeated forms of bullying had higher scores on the stress and anxiety rating scales compared to those who were not exposed or were occasionally exposed.

- 10.7% of students indicated a significant negative relationship between the frequency of bullying and their relationship with their parents and emotional state.

Chen et al. [33] investigated the bullying phenomenon in Chinese middle schools and found that those who were involved in a bullying incident have a higher tendency to develop symptoms of stress and anxiety. Zhang et al. [34] state that anxiety mediates the dynamics between bullying and parental stress.

Hypothesis 2: If students perceive bullying as frequent and severe, then they will report lower school satisfaction compared to the perception of parents and teachers. The results of the research partially validated this hypothesis, showing a significant difference between students’ perceptions and those of parents and teachers regarding the severity and frequency of bullying. Students who perceived bullying as serious and frequent also reported lower school satisfaction. These differences in perceptions were highlighted by qualitative analyses, which revealed that parents and teachers often underestimate the impact of bullying on pupils.- 69% of pupils reported that the bullying incident made them feel less safe in the school environment, which affects their school satisfaction.

- Although most pupils (59.7%) reported that bullying had no significant impact on their relationship with the school, for a substantial proportion, the impact was negative. Varela et al. (2021) [35] reported a negative link between bullying incidents and school satisfaction, however, they did not find a significant link between bullying victimization and school satisfaction among Chilean adolescents. This highlights the complex and intricate nature of bullying and its effects on the school environment.

Aldridge et al. [36] researched bullying and life satisfaction among 6120 Australian students. They state that life satisfaction is strongly linked to the school climate. They also argue that a school environment that is positive can help reduce the negative effects of bullying. The authors also recommend improving the school climate in order to prevent bullying. Winnaar, Arends & Beku, [37] used data from a 2015 TIMSS research study in South Africa. Their analysis reveals a negative correlation between student satisfaction and bullying. Authors demonstrate that a school environment that is orderly, safe and has less disciplinary issues is connected to lower bullying victimization rates.

### Research question 1

The discrepancies between the opinions of experts, teachers, and parents on bullying highlight the need for a comprehensive and detailed approach that involves all key participants. Intervention programs and initiatives need to be supported by a clear educational framework. Parents and teachers must be educated on the bullying phenomenon’s effects, complexity, and implications. This highlights the need for targeted programs. The goal is to create a positive and healthy learning environment for students in which they can thrive and evolve. Van Aalst et al. (2024) report that teachers who perceive bullying as a serious issue are more likely to intervene and confirm discrepancies in how stakeholders view bullying.

### Research question 2

We found an overwhelming consensus among interviewed subjects regarding our second research question. Both experts and parents reported that those who were involved in a bullying incident are more likely to be excluded and isolate themselves from the rest of the group. This finding highlights the importance of close monitoring of bullying victims and aggressors. Kallman, Han, & Vanderbilt [38] discovered a strong correlation between bullying and social isolation. In their study victims of bullying are described as isolated and without a consistent friend group. They also argue that long-term consequences can affect the individual’s ability to maintain and form healthy relationships. Yu and Zhao [39] also consider social isolation as a risk factor for bullying victimization. Hong [40] confirms the fact that social withdrawal is a risk factor when it comes to bullying victimization. However, it is not an omnipresent effect for every victim.

### Research question 3

The qualitative data collected strongly sustains our third research question. The interviewed subjects expressed a popular perception of insufficient anti-bullying measures. They highlighted the need for stakeholder collaborative efforts to enhance monitoring and awareness programs. Parents advocated for programs that prepare both students and teachers to combat bullying. Subjects also raised concerns about the timely reporting of bullying incidents. They suggest that students need to be monitored more closely. In addition, sanctions and framework for each offense need to be clear. The qualitative research revealed that most parents and teachers consider current prevention and intervention measures insufficient. They called for more rigorous strategies, including awareness-raising programs, teacher training, and direct interventions for the pupils involved. The data collected data expresses the need for more structured and comprehensive anti-bullying measures. Hall and Chapman [41] researched policy implementation efficiency and teacher protection of students. They support our study results by stating that teachers have a pivotal role in bullying prevention. However, they face challenges due to a lack of resources and knowledge. They also found that schools with more resources had an easier time following the implemented anti-bullying policies. Fauzan & Sulaeman [42] argue that existing bullying prevention programs have major deficiencies in SMAN 3 Banjar. Like in our study, teachers highlight the need for comprehensive, context-specific approaches involving all important parties (teachers, parents, and students). Tian et al. [43] highlight the insufficiency of bullying prevention programs in secondary schools in China. They argue that there is a lack of empirical evidence when it comes to measuring the effectiveness of such programs, and they call for a more nuanced and comprehensive approach.

Despite the relevance of the proposed interventions, their implementation within Romanian schools may face several structural barriers. These include limited financial and human resources, insufficient numbers of trained school counselors, and the heavy workload already placed on teachers. Additionally, disparities between schools in terms of institutional capacity and access to support services may hinder the consistent application of anti-bullying policies. Acknowledging these constraints is essential, as effective prevention strategies must be adapted to existing resources and supported by sustained institutional commitment.

## Conclusion

Our research has provided an in-depth understanding of the phenomenon of bullying in secondary schools in Craiova, confirming the proposed hypotheses and highlighting the need for more effective measures for prevention and intervention in cases of bullying. The study emphasized the importance of the involvement of all educational actors – students, parents, teachers, and school administration – in combating this complex and dangerous phenomenon.

The results can serve as a basis for developing policies and programs to promote a safe and inclusive school environment for all students. We, the authors, found that there is a difference in perceptions when it comes to bullying, meaning that there needs to be multiple intervention initiatives that involve collaborations with all stakeholders.

In addition, based on the results presented in the previous, we have the following recommendations to enhance existing bullying prevention programs:

- Education and awareness: Conducting activities at school that raise awareness and promote positive social emotions such as empathy, respect, conflict management, and the presentation of the negative effects of bullying.
- Clear policies and consequences: Developing and implementing clear anti-bullying policies in each school, and more severe sanctions for aggressors.
- Training teachers: Organizing courses, training, and sharing useful materials with teachers can have a major impact on how teachers manage and handle bullying incidents. This training should contain efficient strategies for intervention.
- Adopting a report and intervention system: This system could ensure confidentiality but also efficient and timely intervention when a bullying incident occurs.
- Monitorization and evaluation: Each of the implemented programs should be closely monitored and evaluated, then adapted to student’s needs. The goal is to create a safer learning environment for students, and because bullying has long-lasting effects, we could prevent future emotional and psychological issues.

In conclusion, the research contributes to a deeper understanding of the phenomenon of bullying in the educational environment and provides concrete recommendations for improving interventions and prevention policies. Implementing these recommendations can help reduce the incidence of bullying and improve the academic climate, thus promoting the well-being and harmonious development of all students. Further research should focus on comparing these results with schools from other parts of the country. In this manner, we could have a comprehensive understanding of the bullying phenomenon in Romania. In addition, a longitudinal study focusing on the effects of bullying in the long term would provide valuable insight into the long-lasting implications of the bullying phenomenon, beyond describing a short-term context.

## Patents

Informed Consent Statement: Informed consent was obtained from all subjects involved in the study.
